# Fetal Alcohol Syndrome: Does Alcohol Withdrawal Play a Role?

**Published:** 1998

**Authors:** Jennifer D. Thomas, Edward P. Riley

**Affiliations:** Jennifer D. Thomas, Ph.D., is a research assistant professor and Edward P. Riley, Ph.D., is a professor and the director of the Center for Behavioral Teratology, Department of Psychology, San Diego State University, San Diego, California

**Keywords:** fetal alcohol syndrome, AOD withdrawal syndrome, symptom, gestation, fetus, neonate, mother, prenatal alcohol exposure, heavy AOD use, binge AOD use, congenital anomaly, fetal development, central nervous system, NMDA receptors, cytolysis, cell growth and differentiation, teratogenesis, brain damage, AOD abstinence, animal model, literature review

## Abstract

Alcohol use by a pregnant woman may interfere with the development of her fetus. Newborns whose mothers are intoxicated during delivery can experience withdrawal symptoms, such as tremors and even seizures. It is likely that withdrawal also can occur during fetal development. Thus, the possibility exists that withdrawal by the pregnant woman may exacerbate alcohol’s adverse effects on her fetus. One potential mechanism through which alcohol withdrawal might damage the fetus involves the receptor for the neurotransmitter glutamate (i.e., the *N*-methyl-d-aspartate [NMDA] receptor). This receptor plays a crucial role during neuronal development. Excessive activation of the NMDA receptor, which occurs during withdrawal, may lead to neuronal cell death. Animal studies suggest that these effects may contribute to behavioral deficits following prenatal exposure to alcohol.

A woman who drinks alcoholic beverages during pregnancy exposes not only herself but also her fetus to alcohol. Alcohol readily crosses the placenta; consequently, the blood alcohol levels (BAL’s) of the fetus are similar to those of the mother. Likewise, if the woman suddenly abstains from alcohol, both she and her fetus may undergo withdrawal. This article briefly describes the withdrawal symptoms observed in pregnant women and newborns, discusses the birth defects associated with prenatal alcohol exposure, and explores the possibility that alcohol withdrawal (AW) may contribute to the adverse effects of prenatal alcohol exposure on fetal brain function and behavioral development.

## Withdrawal Symptoms in Pregnant Women and Newborns

### Pregnant Women

Despite warning labels on alcoholic beverages and an increased awareness among the general population of alcohol’s deleterious effects on the developing fetus, the women who are the heaviest alcohol abusers frequently do not change their drinking practices during pregnancy. Such women are at the greatest risk for giving birth to a child with alcohol-related problems. In the United States, approximately 3.3 percent of pregnant women are estimated to consume two or more drinks per day (Abel 1990).

One of the consequences of heavy alcohol use is the potential for experiencing withdrawal symptoms during periods of abstinence. Symptoms of mild to moderate withdrawal from alcohol may include tremors, sweating, stomach pain, anxiety, and sleep disturbances. Severe withdrawal symptoms may include delirium tremors and violent agitation (i.e., seizures). These effects may begin within hours of the last drink and may persist for several days. Various tranquilizers and sedatives, including alcohol itself, have been used to manage these symptoms (Thorp 1995).

### Newborns

When a pregnant woman undergoes AW, so does her fetus. To date, no studies have analyzed the symptoms and effects of withdrawal on fetuses in utero. However, researchers have studied withdrawal in newborns whose mothers were intoxicated during delivery. For example, Beattie (1986) reported the case of a newborn who had a BAL of more than 200 milligrams per deciliter (mg/dL) (i.e., over 0.2 percent^1^) and exhibited signs of AW—including tremors, irritability, frequent mouth movements, and vomiting—between 24 and 48 hours after delivery.

The onset of withdrawal symptoms in newborns may be delayed compared with adults, because alcohol metabolism in newborns is slower than in adults. Neonatal AW typically manifests itself as hyperexcitability of the central nervous system (CNS) and gastrointestinal symptoms. CNS hyperexcitability results in symptoms such as tremors, excessive muscle tension, irritability, increased respiratory rate, poor sleeping patterns, and increased sense of hearing (i.e., hyperacusis). These infants also may exhibit spontaneous seizures accompanied by cessation of breathing (i.e., apnea) and arching of the back (i.e., opisthotonos). Gastrointestinal symptoms may include abdominal distention and, in a few cases, vomiting (Robe et al. 1981; see also Coles et al. 1984).

Newborns undergoing AW are best treated by being placed in a calm environment with decreased sensory stimulation. Pharmacological treatment with sedatives or tranquilizers has been used only for infants with the most serious symptoms (e.g., seizures and vomiting).

The observations described here leave little doubt that newborns can undergo AW, and similar effects may occur in the fetus. The effects of maternal or fetal withdrawal (and its treatment) on the developing fetus, however, remain unknown.

## Consequences of Prenatal Alcohol Exposure

Women who drink alcohol during pregnancy place their fetuses at risk for numerous developmental problems, ranging from prenatal mortality to disruptions in physical and behavioral development. The most serious consequence of heavy maternal drinking is a cluster of characteristic anomalies termed fetal alcohol syndrome (FAS) (Jones and Smith 1973). Symptoms of FAS include prenatal and postnatal growth retardation, characteristic facial abnormalities, and CNS anomalies. The distinct facial characteristics of children with FAS include such features as a flat midface, thin upper lip, and small eye openings (i.e., palpebral fissures). CNS anomalies associated with FAS include an abnormally small head (i.e., microcephaly) and behavioral problems, such as attention deficits, hyperactivity, motor dysfunction, mental retardation, and learning and social skills deficits. Although some of the physical characteristics of FAS become less pronounced as the child matures into adulthood, many of the behavioral problems persist.

Even if they do not meet all the criteria for a diagnosis of FAS, children exposed to alcohol in utero may exhibit a wide range of developmental problems, particularly behavioral disorders. These developmental problems often are referred to as fetal alcohol effects or alcohol-related birth defects. The identification of the factors that place a fetus at risk for the harmful effects of prenatal alcohol exposure and the elucidation of the mechanisms by which alcohol causes CNS dysfunction and subsequent behavioral alterations are among the challenges that researchers currently face.

## Can Alcohol Withdrawal Damage the Fetus or Newborn?

There is little doubt that alcohol interferes with normal fetal development (i.e., is a teratogenic agent). Fetal alcohol exposure can adversely affect numerous developmental processes, such as the multiplication (i.e., proliferation), migration, and survival of cells, as well as the cells’ development into specific cell types (i.e., differentiation). The mechanisms by which alcohol disrupts these processes have yet to be fully elucidated. Given alcohol’s ubiquitous distribution throughout all body regions, however, it likely interferes with development through many direct and indirect actions (for a review, see West et al. 1994).

Like alcohol itself, withdrawal from alcohol initiates a cascade of physiological events that might also affect the developing organism. Although there is no direct evidence that withdrawal contributes to alcohol’s adverse effects on development, the possibility demands further investigation.

The clearest indications that withdrawal from drugs can have long-term adverse effects on the developing fetus have come from animal studies examining the teratogenic effects of narcotics. For example, a series of studies evaluating the effects of prenatal opiate exposure have shown that withdrawal contributes considerably to the adverse effects of these agents, increasing mortality and morbidity in fetal and newborn rat pups (for a review, see Sparber 1986). In those studies, pregnant rats (and their fetuses) were exposed throughout gestation to high doses of a long-acting opiate (i.e., 1-alpha-acetylmethadol [LAAM]). Of the rat pups, 50 percent died within 24 hours of birth, a time during which the pups were undergoing withdrawal. This mortality rate decreased to 30 percent when the rats were injected with opiates to alleviate withdrawal. Conversely, injection of naloxone, an agent that increases withdrawal severity, increased the pups’ mortality rate to more than 90 percent. Moreover, when pregnant rats receiving LAAM were injected with naloxone, thereby precipitating withdrawal in both the mother and the fetus, prenatal mortality increased and body weight and length decreased in the offspring, compared with offspring of mothers receiving only LAAM. These observations suggest that opiate withdrawal caused problems independent from narcotic exposure itself and that the severity of these adverse effects was related to the severity of withdrawal.

These studies also indicate that withdrawal can contribute to behavioral alterations associated with early narcotic exposure. For example, rats that have been prenatally exposed to opiates generally are more sensitive to aversive stimuli, such as heat, compared with normal rats. The studies in LAAM-treated rats demonstrated that when postnatal withdrawal was attenuated by injection of opiates during the withdrawal period, the heightened sensitivity to aversive stimuli was mitigated (Sparber 1986).

Just as opiate withdrawal exacerbates the effects of opiate exposure, withdrawal from alcohol might contribute to alcohol’s teratogenicity. To date, only a few studies have examined this possibility. For example, Trofimov and colleagues (1996) found that alcohol-treated rats exhibited more severe cognitive deficits if the alcohol exposure was stopped abruptly than if the alcohol levels were tapered off slowly. Because withdrawal would have been more severe following the abrupt removal of alcohol than following its gradual reduction, these observations suggest that the cognitive deficits were at least in part affected by withdrawal.

In addition, both animal and human studies suggest that maternal consumption of a large amount of alcohol in a short period of time (i.e., binge drinking) produces more severe brain damage and behavioral alterations in the developing organism compared with more chronic alcohol consumption. In animals, for example, a lower dose of alcohol consumed in a bingelike manner that produces a high peak BAL causes more severe microencephaly, neuronal cell loss, and behavioral deficits in the offspring than does a higher daily dose that is administered throughout the day and produces a continuous but lower BAL (Goodlett and West 1992). Similarly, maternal binge drinking in humans produces more severe disruptions in brain electrical wave patterns (i.e., the electroencephalogram [EEG]) in the offspring than does continuous drinking (Ioffe and Chernick 1988). Maternal binge drinking also predicts cognitive variables (e.g., IQ, attention, vigilance, and academic achievement) in prenatally exposed children and adolescents to a greater extent than does frequency of drinking (e.g., Streissguth et al. 1990).

Binge drinking produces a cyclical pattern of high BAL’s followed by withdrawal. Accordingly, these results have been used primarily to illustrate the importance of peak BAL’s as a risk factor for alcohol’s effects on the fetus. However, withdrawal—particularly repeated episodes of withdrawal—possibly contributes to the increased severity of alcohol’s effects on children whose mothers were binge drinkers during pregnancy.

Researchers are only beginning to address the question of how withdrawal from alcohol could damage the fetus or newborn. Several mechanisms that are related either to maternal or fetal withdrawal could conceivably play a role in these processes. For example, acute AW is associated with changes in many of the hormones related to stress. The possibility exists that the stress to the mother and/or the fetus associated with a withdrawal episode could damage the fetus. Episodes of stress during the prenatal period can reduce birth weight, disrupt brain development, decrease immune function, and induce behavioral alterations (e.g., hyperactivity and cognitive deficits). These consequences of prenatal stress all are commonly observed following alcohol exposure during gestation (Abel and Hannigan 1995). In addition to this indirect pathway, AW may directly affect the fetal CNS. The following sections explore this possibility. Because only few published studies have examined the contribution of withdrawal to alcohol’s effects on the fetus, this hypothesis remains speculative.

## Alcohol Withdrawal and the NMDA Receptor

Recently, researchers have been increasingly interested in the direct interaction of alcohol with proteins in the membranes of nerve cells (i.e., neurons) that contribute to the symptoms associated with AW. Some of these proteins form receptors, molecules that interact with the chemicals released by neurons for neuronal communication (i.e., neurotransmitters). Activation of these receptors can either excite the cell, making it more likely to transmit information to other neurons, or inhibit the cell, making it less likely to transmit information. One receptor in particular has received much attention in the analysis of alcohol’s effects on the brain. This receptor, which interacts with the neurotransmitter glutamate—an amino acid that excites certain neurons during normal neurotransmission—can also be specifically activated by *N*-methyl-d-aspartate (NMDA) and is therefore referred to as the NMDA receptor. (For more information on the role of neurotransmitters and their receptors, including the NMDA receptor, in AW, see the article by Littleton, pp. 13–24.)

In adult rats, acute alcohol treatment inhibits NMDA receptors, thereby producing an overall inhibitory effect. With continued alcohol exposure, however, the CNS attempts to offset this inhibitory effect by increasing the number and/or activity of NMDA receptors. This process is called neroadaptation. During abstinence, as alcohol is eliminated from the body, alcohol’s inhibitory action decreases. As a result, the cells with elevated NMDA receptor levels are much more excited than under normal conditions, a phenomenon called rebound excitability. This hyperexcitability may contribute to the symptoms associated with AW, such as tremors, agitation, and seizures. This hypothesis is supported by findings that agents which block activation of NMDA receptors reduce withdrawal symptoms, whereas agents that activate these receptors exacerbate withdrawal symptoms.

During normal neurotransmission, activation of the NMDA receptor excites the neuron. If this receptor becomes overactivated, however, a cascade of intracellular events may occur, resulting ultimately in cell death. This process is called excitotoxicity. Excitotoxicity may result from several different types of insult, including lack of oxygen (i.e., hypoxia), interrupted blood supply (i.e., ischemia), low blood sugar levels (i.e., hypoglycemia), and epilepsy. Excitotoxicity also may occur in several chronic neurodegenerative diseases, such as Alzheimer’s disease and Huntington’s disease (for a review, see Choi 1992).

NMDA receptor-mediated excitotoxicity also may occur during AW, possibly leading to cell loss in various brain regions, including the cortex, hippocampus, and striatum^2^ (Lovinger 1993). This hypothesis is supported by the observation that withdrawal symptoms during abstinence occur at the same time at which glutamate levels in the brain rise and their interaction with the NMDA receptor increases. Experiments both on isolated neurons and on intact brains have demonstrated that during this period, the neurons are more vulnerable to glutamate- or NMDA-induced excitotoxicity. For example, Davidson and colleagues (1995) have demonstrated that hippocampal cells, which are rich in NMDA receptors, are more sensitive to NMDA-induced damage in subjects undergoing withdrawal than in control subjects. Studies using isolated neurons from the cerebellum^3^ and the cortex also have demonstrated that NMDA’s excitotoxic effects on these cells are enhanced during alcohol withdrawal (for a review, see Hoffman and Tabakoff 1994). Finally, slices of brain tissue from the hippocampus that are intermittently exposed to alcohol are more sensitive to NMDA-induced excitotoxicity compared with slices continuously exposed to alcohol (Becker 1996). The most severe excitotoxic effects were observed in slices that had undergone the greatest number of “withdrawal” episodes, a phenomenon called kindling (for more information on kindling, see the article by Becker, pp. 25–33).

### The NMDA Receptor in Fetal Development and Alcohol Withdrawal

Mechanisms similar to those described in the previous section may play a role in alcohol’s effects on the fetus, particularly because the developing organism is more vulnerable to NMDA receptor-related excitotoxicity compared with the adult. This vulnerability appears to result from the role that NMDA receptor activation plays during neuronal development (for a review, see McDonald and Johnston 1990). Both NMDA and glutamate promote neuronal growth and regulate the formation of connections among neurons (i.e., neuronal circuits). For example, NMDA receptor activation has the following effects:

- Promotes survival of the neuron whose receptors are activated
- Stimulates the outgrowth of dendrites, the branchlike extensions of neurons where signals from other neurons are received
- Affects the pattern of dendrite branching
- Influences the formation of synaptic connections that allow neurons to communicate with each other.

Through these functions, NMDA receptors play a critical role in the ability of neurons to alter their structure or function (i.e., neuronal plasticity), a process that is essential for brain development.

During periods of neurite outgrowth and synapse formation, the number of NMDA receptors on many neurons increases for a short period of time. For example, in the rat hippocampus, NMDA receptor levels between 6 and 14 days after birth are at 150 to 200 percent of the adult level (Tremblay et al. 1988; for a review, see also Hattori and Wasterlain 1990). A similar transient increase in NMDA receptor levels occurs in the hippocampal formation of the human fetus between 23 and 27 weeks of gestation, and the density of NMDA receptors in human newborns is higher than in adults (Retz et al. 1996).

The increase in NMDA receptor levels also enhances the neurons’ vulnerability to glutamate’s excitotoxic action. For example, the susceptibility of rat pups to NMDA receptor-mediated excitotoxicity rises dramatically after birth, peaking at postnatal days 6 and 7. At the same time, the animals become more vulnerable to alcohol-related disruptions in the development of behaviors that require the functional integrity of the hippocampus, which is rich in NMDA receptors. Moreover, on postnatal day 6, rats become more vulnerable to alcohol-induced weight reductions of the forebrain, which also is rich in NMDA receptors. Thus, the vulnerabilities to alcohol and NMDA-related insults coincide during development, suggesting that NMDA receptor-associated mechanisms may contribute to alcohol’s effects on the fetus.

During fetal development, NMDA receptor activity must be tightly controlled to maintain a balance between the receptor’s neuronal growth-promoting effects and its excitotoxic effects. Accordingly, both underactivation and overactivation of this receptor could be damaging to the developing organism. Thus, alcohol exposure could disrupt neuronal development by directly inhibiting NMDA receptors, whereas AW could interfere with development by over-activating NMDA receptors. For example, in studies using cultured cells, Pantazis and colleagues (1995) have demonstrated that the addition of NMDA to cerebellar cells in the presence of alcohol protected against alcohol-induced cell death (see also Reynolds and Brien 1995). These findings indicate cell death could be prevented by activating the NMDA receptor during alcohol treatment.

**Figure 1:**
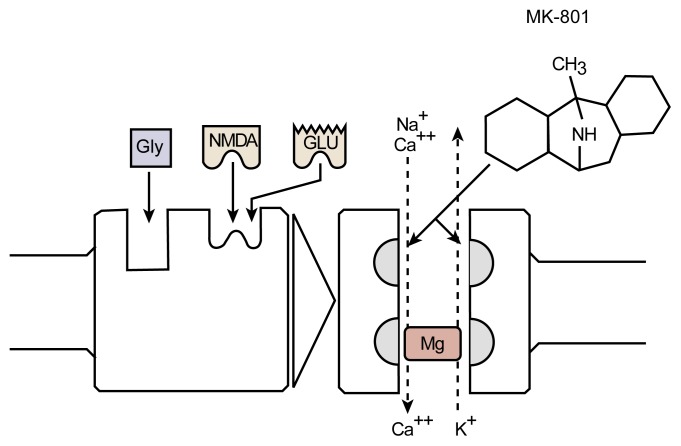
The NMDA receptor complex. Activation (i.e., excitation) occurs when either glutamate (Glu) or *N*-methyl-d-aspartate (NMDA) and glycine (Gly) bind to the receptor molecule. A channel within the receptor complex enables molecules to cross the cell membrane. Magnesium (Mg) blocks this channel. When Mg is removed from the channel and the receptor is activated, calcium (Ca^++^) and sodium (Na^+^) ions enter the cell and potassium ions (K^+^) leave. MK-801 prevents the flow of ions across the membrane by binding to a site within the ion channel, thereby blocking NMDA receptor function and protecting the cell against excess activation (i.e., excitotoxicity).

Thomas and colleagues (1997) investigated the relationship between the NMDA receptor, AW, and the severity of alcohol-related behavioral deficits. In their study, rat pups were exposed to a high level of alcohol on postnatal day 6, a period of brain development that is equivalent to a portion of the human’s third trimester of pregnancy. During the withdrawal phase, the animals were injected either with saline or MK-801, a substance that blocks the NMDA receptor and mitigates excitotoxic cell death (see figure 1). Approximately 5 weeks later, the animals were tested on a spatial reversal learning task. For this test, the animals first learned that they could escape from a water maze in only one of two locations. The investigator then reversed the location where the escape was afforded and recorded how often the animals went to the wrong location before they found the new escape location. The animals’ performance on this task was shown to be impaired by early alcohol exposure as well as by damage to the hippocampus and cortex, two areas that are vulnerable to excitotoxic cell death.

**Figure 2:**
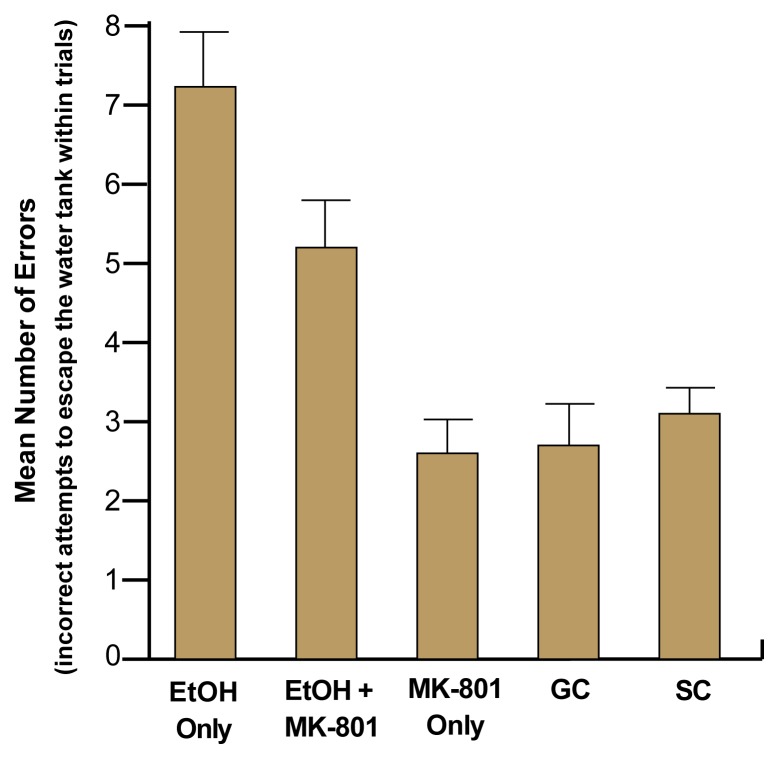
Administration of MK-801 during neonatal withdrawal reduced alcohol’s teratogenic effects on learning behavior. Researchers administered MK-801 to neonatal animals after treating them with alcohol to see if it would affect the rats’ ability to perform a learning task (i.e., to find the escape route out of a water tank) later in life. During withdrawal from alcohol, some of the animals were given MK-801 (EtOH + MK-801). For comparison, other animals were given only alcohol and no MK-801 (EtOH Only), were given only MK-801 but no alcohol (MK-801 Only), or were not treated with anything at all (GC and SC). Of the five groups, the EtOH Only group committed the greatest number of errors, showing difficulty in switching from the incorrect to the correct location within the maze. Alcohol-exposed rats treated with MK-801 during the withdrawal period (EtOH + MK-801) did not do as well as the control rats but did much better than the EtOH Only group. MK-801–treatment alone did not significantly affect performance. SOURCE: The results presented here have been simplified for presentation purposes. For details on the methodology used in this experiment, see Thomas et al. 1997.

As expected, performance on this task was impaired in animals that had been exposed to alcohol compared with control animals. However, those animals that had been exposed to alcohol and treated with MK-801 during withdrawal performed significantly better than did animals that had been treated with saline during withdrawal (see figure 2). Administration of MK-801 alone had no effect on any behavioral measure. These findings indicate that the severity of alcohol-related behavioral deficits can be significantly reduced by blocking NMDA receptors during withdrawal, thereby reducing the severity of the withdrawal.

These data support the contention that excitotoxicity may occur during episodes of withdrawal in the developing organism and may contribute to alcohol’s detrimental effects on the fetus. However, more direct evidence of the effects of alcohol exposure and withdrawal on NMDA receptors and glutamate release during development is needed. For example, the study by Thomas and colleagues (1997) examined only the effects of acute alcohol administration and may not reflect events occurring during more prolonged exposure, which may be required to initiate withdrawal in humans. Some studies have indicated, however, that withdrawal may be related to total alcohol exposure and that even one-time alcohol exposure can induce a regular withdrawal response if the alcohol dose is high enough (Goldstein 1986). Obviously, more studies involving various models of alcohol exposure are necessary to resolve these issues.

## Conclusions

Prenatal alcohol exposure can have devastating effects on the growth and development of the fetus. Although alcohol can disrupt development through many mechanisms, both direct and indirect, little research has addressed the issue of whether withdrawal contributes to alcohol’s deleterious effects. Researchers are now beginning to understand the role of neurotransmitter receptors in excitotoxic brain damage and in the development of withdrawal symptoms. The consequences of alcohol’s actions at these receptors are highly complex in the dynamic context of development, because the neurotransmitters that react with these receptors also play important roles in neural development. For example, NMDA receptor activation can result in both neuronal growth-promoting and excitotoxic effects, and its activity therefore must be finely balanced. Consequently, both inhibition of the NMDA receptor after alcohol exposure and activation of the receptor during withdrawal could interfere with neuronal development. Future research on the direct and indirect effects of withdrawal on the fetus and newborn may result in more effective treatment strategies to reduce the long-term consequences of prenatal alcohol exposure.

## Footnotes

1 In most States, the legal BAL for driving ranges from 0.08 to 0.1 percent.

2 The cortex, the outer layer of neurons covering the brain, contains areas for processing sensory information and controlling motor functions, speech, and higher cognitive functions. The hippocampus is a brain region thought to play a role in learning and memory. The striatum is a brain region below the cortex that is thought to be involved in motor function and some cognitive functions.

3 The cerebellum is a brain region involved in motor control.
